# Multi-system benefits of non-invasive spinal cord stimulation following cervical spinal cord injury: a case study

**DOI:** 10.1186/s42234-025-00183-8

**Published:** 2025-09-05

**Authors:** Soshi Samejima, Claire Shackleton, Raza N. Malik, Ali Hosseinzadeh, Lucas Rempel, Anh-Duong Phan, Alison Williams, Tom Nightingale, Amandeep Ghuman, Stacy Elliott, Matthias Walter, Klaus Krogh, Michael Berger, Tania Lam, Rahul Sachdeva, Andrei V. Krassioukov

**Affiliations:** 1 International Collaboration on Repair Discoveries (ICORD), University of British Columbia, Vancouver, BC Canada; 2 Division of Physical Medicine and Rehabilitation, Faculty of Medicine, University of British Columbia, Vancouver, BC Canada; 3 School of Kinesiology, University of British Columbia, Vancouver, BC Canada; 4 School of Sport, Exercise and Rehabilitation Sciences, University of Birmingham, Birmingham, UK; 5 Department of Surgery, St. Paul’s Hospital, Vancouver, BC Canada; 6 Department of Psychiatry, University of British Columbia, Vancouver, BC Canada; 7 Faculty of Medicine, University of Basel, Basel, Switzerland; 8 Department of Clinical Medicine, Department of Hepatology and Gastroenterology, Aarhus University Hospital, Aarhus, Denmark; 9 GF Strong Rehabilitation Centre, Vancouver Coastal Health, Vancouver, BC Canada; 10 Department of Physical Medicine & Rehabilitation, University of Kentucky, Lexington, KY USA; 11 Spinal Cord and Brain Injury Research Center, University of Kentucky, Lexington, KY USA; 12 GF Strong Rehabilitation Centre, University of British Columbia ICORD-BSCC, UBC, 818 West 10th Avenue, Vancouver, BC V5Z 1M9 Canada; 13 Division of Physical Therapy, Department of Rehabilitation Medicine, University of Washington, 1959 NE Pacific St, Seattle, WA 98195 USA

**Keywords:** Spinal cord injury, Transcutaneous spinal cord stimulation, Autonomic dysfunction, Motor function

## Abstract

Cervical spinal cord injury (SCI) impairs sensorimotor and autonomic functions. We investigated the effects of lumbosacral transcutaneous spinal cord stimulation (tSCS) on urinary bladder, bowel, and sexual function as well as cardiovascular and sensorimotor functions in one individual with chronic clinically motor-complete cervical SCI, 11 years post-injury. Following 30 sessions of lumbosacral tSCS, the individual presented with improved urinary bladder compliance as well as anorectal function in parallel with mitigation of the severity of autonomic dysreflexia during filling cystometry and anorectal manometry. In addition, long-term tSCS improved erectile function and sexual satisfaction. Furthermore, after long-term tSCS, concurrent tSCS enabled voluntary leg movement. This case study demonstrated the multi-system benefits of lumbosacral tSCS, which holds promise for evolving into a clinically viable therapy for restoring both autonomic and motor functions following SCI.

**Supplementary Information:**

The online version contains supplementary material available at 10.1186/s42234-025-00183-8.

## Introduction

Spinal cord injury (SCI) results in both sensorimotor and autonomic dysfunctions, negatively impacting quality of life. Cervical SCI, the most common form of traumatic SCI (NSCISC 2024), is particularly detrimental as it disrupts communication between the brain and spinal circuits accountable for all extremities and autonomic functions. The prevalence of autonomic dysfunctions, including lower urinary tract (LUT), bowel, and sexual complications, alongside blood pressure (BP) dysregulation, is notably high in people with cervical SCI (Ng et al. 2005; Weld and Dmochowski 2000; Anderson et al. 2007; Wecht et al. 2021). Furthermore, pelvic organ dysfunction after SCI is often accompanied by abnormal cardiovascular responses such as autonomic dysreflexia (AD) (Krassioukov 2009). Improving autonomic functions is among the highest recovery priorities for individuals with SCI (Anderson 2004; Wheeler et al. 2018; Simpson et al. 2012; Garshick et al. 2005). However, current pharmacological and non-pharmacological interventions for managing autonomic dysfunctions remain insufficient. Current therapeutic approaches often focus on a single function, have significant side effects, and involve invasive procedures (Ginsberg et al. 2021; Johns et al. 2021; Medicine CfSC 2010).

Recent evidence demonstrates that invasive epidural spinal cord stimulation can improve motor (Rowald et al. 2022; Gill et al. 2018; Angeli et al. 2018) and autonomic (Samejima et al. 2023; Harkema et al. 2018; Squair et al. 2021; Herrity et al. 2022; Shackleton et al. 2023) functions in individuals with SCI. In contrast to invasive surgical approaches, transcutaneous spinal cord stimulation (tSCS) offers more flexibility for targeting multiple spinal cord segments without surgery by adjusting the electrode montage placed over the skin (Taccola et al. 2020). Electrophysiological and computational modeling evidence indicates that tSCS activates similar neural structures and large-diameter afferent fibers, as observed with epidural spinal cord stimulation (Capogrosso et al. 2013; Hofstoetter et al. 2018). According to epidural spinal cord stimulation studies, the inputs through afferent fibers could likely activate spinal interneurons, motoneurons, and preganglionic neurons (Squair et al. 2021; Wagner et al. 2018). Similar to epidural spinal cord stimulation, tSCS over the lumbosacral spinal cord can improve autonomic and sensorimotor functions in people with SCI (Kreydin et al. 2020, 2022; Samejima et al. 2022a, b). The dual benefits of lumbosacral tSCS are likely due to the overlap of the autonomic and somatosensory neural pathways in the lumbosacral spinal cord (Wecht et al. 2021; Rupp et al. 2021). A recent review has highlighted the potential multi-system benefits of spinal cord stimulation following SCI (Moreno Romero et al. 2024), however, no study has systematically quantified improvements across multiple organ systems in the same individual. This manuscript presents a case study from an ongoing clinical trial (NCT04604951). We evaluated the effect of 30 tSCS sessions on LUT, bowel, and sexual functions as well as cardiovascular effects using quantitative methods in one individual with chronic clinically motor-complete cervical SCI. We also evaluated potential effects on lower extremity motor function after the intervention.

## Methods

**Fig. 1:**
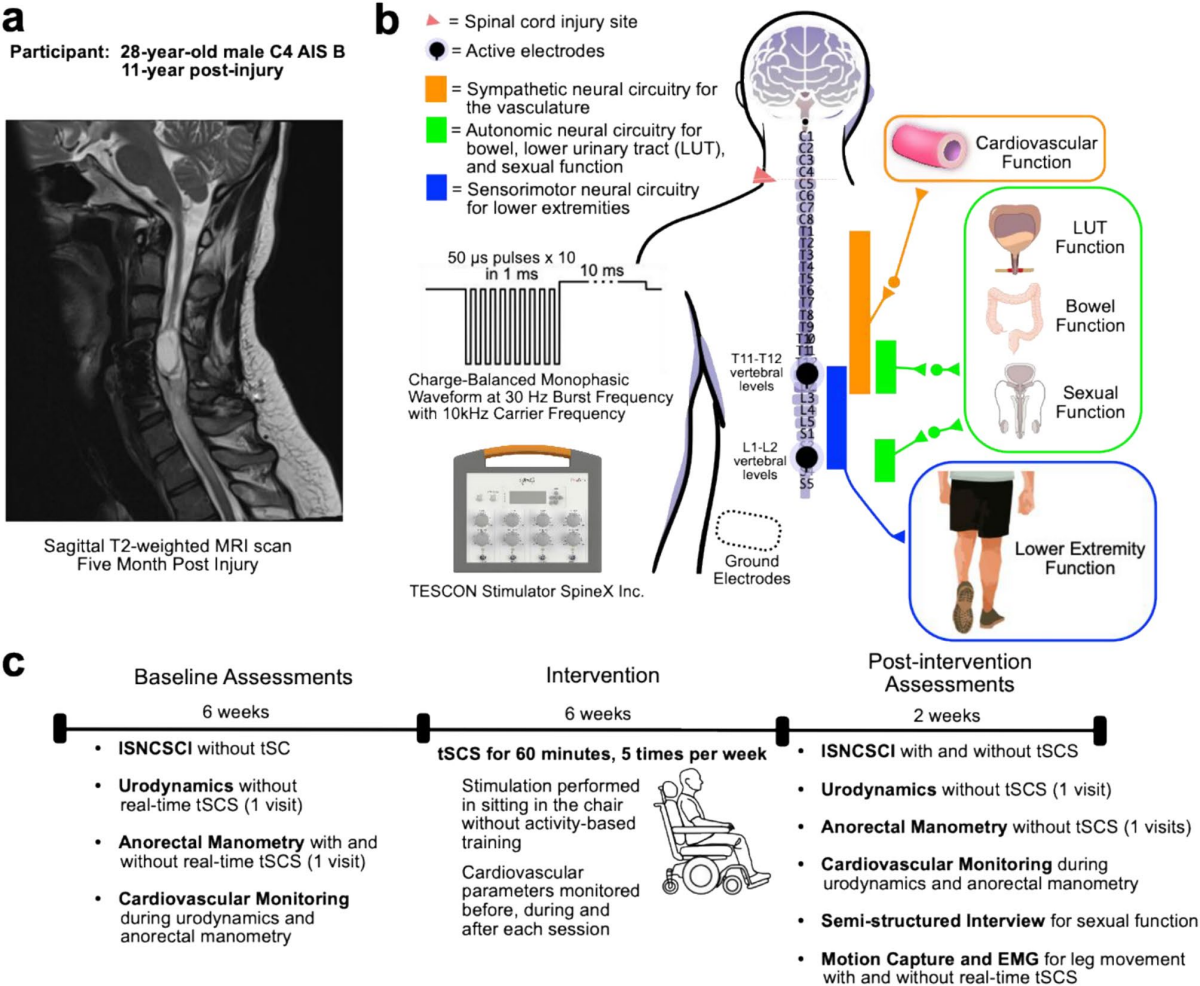
Participant Information and Study Intervention. **(a)** Sagittal T2-weighted MRI scan showing a near complete transection of the spinal cord at C5-C6 levels with the intervening cord parenchyma demonstrating cystic degeneration and myelomalacia. **(b)** Transcutaneous spinal cord stimulation was delivered through two active electrodes at T11-T12 and L1-L2 vertebral levels to activate the lumbosacral spinal cord involved in control of the peripheral vasculature, pelvic organs, and lower extremity muscles. Charge-balanced monophasic 1 ms pulses at 30 Hz with 10 kHz carrier frequency were delivered in a seated position without activity-based training. **(c)** Study design and timeline with pre- and post-intervention assessments. Abbreviation: AIS = American Spinal Injury Association Impairment Scale, C = Cervical, L = Lumbar, LUT = Lower Urinary Tract, ISNCSCI = International Standards for Neurological Classification of Spinal Cord Injury, T = Thoracic, tSCS = transcutaneous spinal cord stimulation, EMG = Electromyography

A 28-year-old male with a traumatic SCI classified as motor-complete, American Spinal Injury Association Impairment Scale (AIS) B, with a neurological level of injury at the fourth cervical spinal segment (C4) was recruited for this study 11 years post-injury. The injury occurred in 2012 when a tree fell on his neck, for which he underwent anterior spinal fusion and stabilization. A sagittal T2-weighted MRI showed a near complete transection of the spinal cord at C5-C6 level (Fig. 1a). The participant provided informed consent for this study, including all assessments and interventions that were approved by the University of British Columbia institutional review board (CREB H20-01163).

### Study design

An overview of the experimental procedure for the clinical trial is detailed in our published protocol (Samejima et al. 2022a, b). Baseline assessments of neurological and pelvic organ functions were conducted, including the International Standards for Neurological Classification of Spinal Cord Injury (ISNCSCI), filling cystometry, anorectal manometry, and validated questionnaires for autonomic functions. Filling cystometry and anorectal manometry were conducted without tSCS to investigate their baseline function, while also monitoring cardiovascular responses. Anorectal manometry was performed with tSCS to test the immediate effect of concurrent tSCS. Following these baseline assessments, the participant was randomly assigned to receive 60 min of tSCS in a seated position, five times per week for six weeks, totaling 30 sessions (long-term tSCS). Post-intervention assessments performed without tSCS comprised the same assessment set as the baseline measurements, a semi-structured interview to evaluate sexual function, and neurophysiological and motion capture recordings to assess voluntary motor function with and without concurrent tSCS (Fig. 1c).

### Intervention

tSCS was delivered using a Health Canada-approved (Application#336738) experimental portable spinal cord stimulator (TESCoN, SpineX Inc., USA) (Kreydin et al. 2020). Cathodal stimulation electrodes (1.25” Valutrode round electrode, Axelgaard, USA) were placed between the eleventh and twelveth thoracic vertebral levels (T11-T12) as well as the first and second lumbar vertebral levels (L1-L2) (Fig. 1b). Two anodal electrodes (2” x 3” Valutrode rectangular electrode, Axelgaard, USA) were positioned bilaterally over the posterior iliac crests. During the 60-minute intervention sessions, the stimulator delivered a charge-balanced monophasic waveform consisting of 1 ms pulse width with an overlapping 10 kHz carrier frequency at 30 Hz (Fig. 1b) (Malik et al. 2024). Stimulation current intensity was set to motor subthreshold for lower extremity muscles and reached a maximum of 130 mA. Stimulation parameters and location were determined based on previous studies for motor and pelvic organ functions in individuals with SCI (Kreydin et al. 2022; Samejima et al. 2022a, b; Moritz et al. 2024; Tefertiller et al. 2021). During the intervention sessions, cardiovascular responses were monitored using a brachial BP monitoring device (Dynamap Pro, GE Healthcare, USA) for safety.

### Evaluation of lower urinary tract function

Filling cystometry was performed using the Aquarius-TT Model 94-R03-BT (Laborie, USA) in accordance with Good Urodynamic Practices recommended by the International Continence Society (Schäfer et al. 2002) to assess LUT function. We assessed and recorded the first sensation of urinary bladder filling (mL), strong desire to void / empty urinary bladder (mL), presence of neurogenic detrusor overactivity (NDO), volume at the first NDO (mL), change (Δ) in detrusor pressure until first NDO (mL), maximum cystometric capacity (mL), maximum Δ in detrusor pressure (cmH_2_O), urinary bladder capacity (= t-void residual (mL), urinary bladder compliance (mL/cmH_2_O), and presence of urinary leakage. The participant underwent filling cystometry in a supine position during the baseline visit and post-intervention visit. Prior to each LUT assessment visit, the participant refrained from taking Myrbetriq™ (a beta-3 adrenergic receptor agonist) 25 mg per day, which the participant took daily, for one week and was asked to empty his urinary bladder.

Urinary bladder ultrasound (Cubescan BioCon-700, Roxon, Canada) was used to assess urine volume prior to each filling cystometry. Then, the urinary bladder was completely emptied using a catheter prior to each filling cystometry. Urinary bladder volume was always noted (i.e. urine volume obtained using a catheter following each filling cystometry). A transurethral double-lumen catheter (7 Fr, Laborie, USA) was inserted to facilitate retrograde urinary bladder filling with body-warm (37 °C) isotonic saline solution at a filling rate of 30 mL/min to record intravesical pressure. A catheter (9 Fr, Laborie, USA) was inserted into the rectum to record intraabdominal pressure. Detrusor pressure was calculated by subtracting intraabdominal pressure from intravesical pressure. Surface electromyography (EMG) electrodes were placed close to the perineum to record external urethral sphincter and pelvic floor muscle activity. Prior to the start of each filling cystometry and after every 100 mL of infused saline, the participant was asked to cough to confirm the correct placement and recording of all catheters. During filling cystometry, the participant was asked to report his first sensation of urinary bladder filling and strong desire to void / empty his urinary bladder.

During the baseline LUT assessment visit, filling cystometry was conducted without tSCS. After the completion of 30 intervention sessions, filling cystometry was repeated without tSCS during the post-intervention visit. Throughout each filling cystometry, changes in BP were carefully monitored as detailed below. Furthermore, we used the Neurogenic Bladder Symptom Score (NBSS) (Welk et al. 2014) as a patient-reported outcome measure for assessing changes in LUT function at baseline and after 30 sessions of tSCS.

### Evaluation of bowel function

To assess physiological changes in anorectal function, high-resolution anorectal manometry was performed using a high-resolution anorectal probe, consisting of a balloon-tipped solid-state catheter with 16 circumferential regions including 256 total sensors (Manoscan, Medtronic, USA) (Aloysius et al. 2023), with the participant in the left decubitus position. The anorectal manometry study, conducted by a colon surgeon, was performed at baseline and after the completion of 30 tSCS sessions. The participant was asked to fast for at least six hours and perform an enema prior to each procedure.

An anorectal examination was performed prior to anorectal manometry in order to examine the perianal skin, the anal canal, and the rectum to check for any external hemorrhoids. Subsequently, the manometry probe was inserted into the anorectal region, with the balloon situated in the distal rectum, 12 cm distal to the anal verge. Anorectal manometry study recorded resting pressure, squeeze pressure, rectoanal inhibitory reflex, and anorectal sensation (Carrington et al. 2020). The mean resting pressure was measured twice at 20-second intervals. Then, the maximum squeeze pressure was measured twice by asking the participant to squeeze their pelvic floor muscles as hard as they could for as long as they could, up to 10 s. To determine the effect of squeeze, the obtained squeeze pressure was subtracted from the resting pressure, and the delta value was reported. The presence, duration, and pressure reduction during rectoanal inhibitory reflex were recorded. The rectoanal inhibitory reflex is the relaxation of internal anal sphincter muscles followed by colon distension, which is important for evacuation (Krogh et al. 2002). The relaxation is mediated by the central nervous system and enteric nervous system (Stebbing et al. 1996). The rectoanal inhibitory reflex was induced by inflating the balloon to 60 mL of air in the distal rectum and recording the changes in the anal canal. The presence of rectoanal inhibitory reflex was determined as a decrease in anal canal pressure of at least 20% compared to the preceding resting pressure (Cheeney et al. 2012). Reflex duration was defined as the point of recovering the anal sphincter pressure to the point up to 80% of the preceding resting pressure. The amplitude reduction of rectoanal inhibitory reflex was calculated as the minimum anorectal pressure and the percentage change in the pressure as it reached the minimum during rectoanal inhibitory reflex compared to the preceding resting pressure. Anorectal sensation was determined by inflating the balloon in increments of 10 mL of air every 10 s up to a maximum of 200 mL. The participant was asked to verbally report the first sensation and the urge to defecate during balloon inflation.

Anorectal manometry during real-time tSCS was carried out to characterize the response of resting pressure to different tSCS intensities during the baseline visit. All anorectal manometry data were analyzed using the ManoView AR 3.0 (Medtronic, USA). Lastly, we used the Neurogenic Bowel Dysfunction Score (NBDS) (Erdem et al. 2017) for assessing changes in daily bowel function at baseline and after 30 sessions of tSCS.

### Evaluation of sexual function

We used the International Index of Erectile Function-15 (IIEF) questionnaire (Rosen et al. 2011) to assess changes in erectile function before and after the intervention. We also conducted a semi-structured interview after the completion of the intervention to assess changes in overall sexual health. An in-depth individual interview was conducted by a sexual medicine physician with extensive clinical and research experience with the SCI population. The semi-structured interview questions, developed by the sexual medicine physician, covered various aspects of sexual function including libido, arousal, erection, ejaculation, orgasm, and pelvic/visceral sensations. Secondary topics such as BP regulation and bowel and bladder function were also addressed. This comprehensive range of topics helped to assess the multifaceted nature of sexual function after SCI. We audio-recorded the interviews and transcribed them verbatim for subsequent analysis using NVivo (Lumivero, USA). The data were analysed to extract the codes and themes. The participant infrequently took phosphodiesterase 5 inhibitors (Cialis™ 5 mg), which was only for sexual activity throughout the protocol.

### Evaluation of cardiovascular function during pelvic organ assessments

Blood pressure (BP) and heart rate responses were monitored during the filling cystometry and anorectal manometry procedures without the application of tSCS. Continuous beat-by-beat BP was measured via finger photoplethysmography (Finapres NOVA, Finapres Medical Systems, Netherlands) in parallel to monitoring minute-by-minute brachial BP (Dynamap Pro, GE Healthcare, USA). Heart rate was also monitored continuously via three-lead electrocardiogram. Baseline BP, maximum systolic BP (SBP), and the severity of AD (noted if a 20 mmHg or more increase in SBP above baseline) (Wecht et al. 2021) were recorded during both filling cystometry and anorectal manometry procedures.

### Evaluation of lower extremity motor function

The 2019 International Standards for Neurological Classification of Spinal Cord Injury (ISNCSCI) examination (Rupp et al. 2021) was conducted by an experienced clinician certified by the American Spinal Injury Association (ASIA) at baseline and after 30 sessions of tSCS. After the intervention phase, we conducted kinematic and surface electromyography (EMG) recordings. The participant was asked to attempt knee extension and ankle dorsiflexion with verbal cues in a seated position. The Optotrak motion capture system was used and recorded at 100 Hz (Northern Digital Inc., Canada). Infrared emitting diodes were placed over the right greater trochanter, lateral epicondyle of the femur, lateral malleolus, and first toe. Kinematic data were processed using a low-pass 4th-order Butterworth filter with a cut-off frequency of 6 Hz. EMG data were recorded with a sampling rate of 2000 Hz (Delsys, USA) from the right rectus femoris, vastus lateralis, adductor magnus, biceps femoris, tibialis anterior, medial gastrocnemius, and soleus muscles based on the recommendations of surface EMG for the non-invasive assessment of muscles (Stegeman and Hermens 2007). EMG data were processed using a bandpass 4th-order Butterworth filter with cut-off frequencies of 10–100 Hz, and a 30 Hz notch filter was applied to reduce tSCS artifacts. Finally, the EMG signals were rectified, and a root mean square (RMS) envelope was created using a 100 ms moving window. We used mean background EMG + 3 standard deviations (SD) as the muscle activation threshold to assess voluntary contraction of each muscle.

### Statistics analysis

All data were summarized using descriptive statistics, including mean values with standard deviation, along with all individual trial data unless noted. When applicable, validated metrics such as the minimal clinically important difference (MCID) or minimal detectable change (MDC) were reported for the outcome measures to provide context for the observed changes. The data analysis was performed using MATLAB R2024a (Mathworks, Natick, MA, USA).

## Results

### Intervention progress

The participant, 28-year-old male with chronic SCI AIS B at C4 level, successfully completed 30 sessions of tSCS, five times per week, for 60 min seated in his power wheelchair (Fig. 1). The average stimulation current intensity was 127 ± 7 mA (100 mA–130 mA). The tSCS therapy was tolerated well without any adverse cardiovascular events. During the intervention sessions, BP and heart rate were safely elevated (Table 1). One minor incident of skin irritation was reported at the site of the cathodal electrodes. This irritation was evaluated by the trial physician and did not impact the protocol.

**Table 1:** Cardiovascular monitoring during 30 intervention sessions

Phase	Pre-session Without tSCS	Mid-session With tSCS	Mid-session Change from baseline with tSCS	Post-session Without tSCS
Systolic Blood Pressure (mmHg)	101 ± 9 [65–114]	109 ± 8 [96–112]	Δ8 ± 14 [-15–59]	106 ± 9 [92–130]
Diastolic Blood Pressure (mmHg)	60 ± 5 [53–73]	63 ± 7 [54–78]	Δ4 ± 8 [-16–25]	59 ± 5 [51–68]
Heart Rate (bpm)	69 ± 9 [54–92]	97 ± 13 [67–119]	Δ28 ± 14 [1–56]	103 ± 14 [80–130]

Note: mean ± standard deviation [min–max] of 30 sessions at baseline without tSCS, mid-session with tSCS, mid-session change in all parameters from baseline with tSCS, and post-session without tSCS

### Lower urinary tract function

**Fig. 2:**
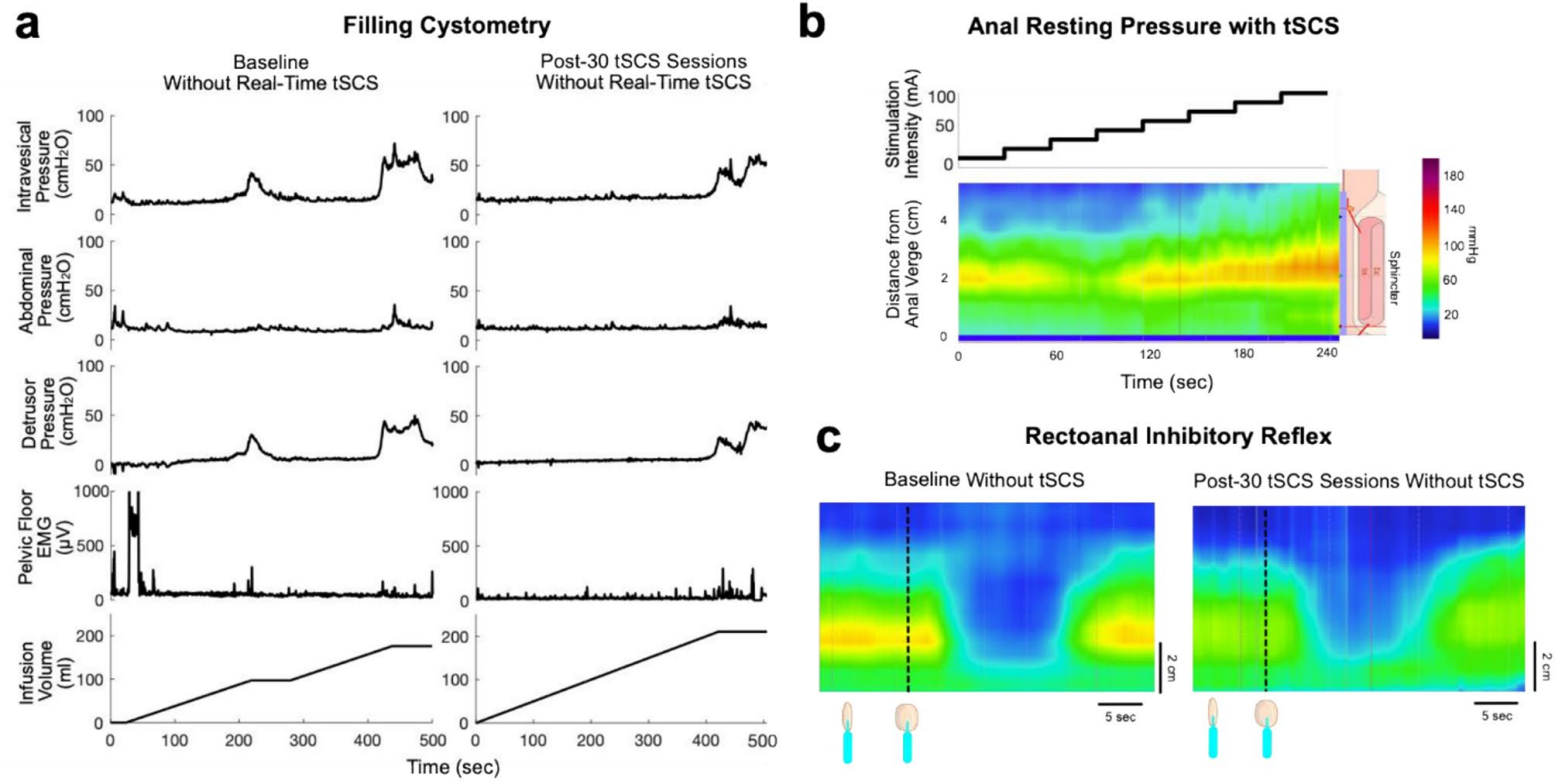
Transcutaneous spinal cord stimulation for lower urinary tract and anorectal functions. **(a)** Filling cystometry was conducted at baseline and after 30 sessions of tSCS. The figure shows the results with NO tSCS. Following the intervention, the participant demonstrated increased urinary bladder compliance. The early onset of detrusor overactivity observed during the baseline assessment was mitigated after long-term tSCS. **(b)** The heatmap color represents anal resting pressure measured by the pressure sensor probe. At baseline, tSCS was applied at T11-T12 and L1-L2 vertebral levels with intensities ranging from 13 mA to 104 mA. **(c)** The left panel shows the recto-anal inhibitory reflex at baseline. After the inflation of the rectal balloon (the dotted line), the anal canal showed relaxation from the resting pressure 111 mmHg to 63 mmHg for 16.5 s. The right panel shows the rectoanal inhibitory reflex after 30 tSCS sessions. In addition to decreased anal resting pressure (73 mmHg), the rectoanal inhibitory reflex induced anal relaxation to 43 mmHg. Abbreviation: tSCS = transcutaneous spinal cord stimulation

During baseline filling cystometry without tSCS, the participant presented with NDO, which was first observed at infused volume of 85 mL. Prior to the onset of NDO, Δ detrusor pressure was 8 cmH_2_O (Table 2; Fig. 2a). The bladder compliance was 10 mL/cmH_2_O. Filling cystometry was paused and restarted since AD did not occur, and detrusor pressure dropped to 2 cmH_2_O after one minute. Terminal NDO occurred at an infused volume of 165 mL. The remaining parameters are highlighted in Table 2; Fig. 2a.

After 30 tSCS sessions, filling cystometry revealed the mitigation of NDO, hence the occurrence of the first appearance of NDO was delayed to an infusion volume of 200 mL. Prior to the onset of NDO, Δ detrusor pressure was 4 cmH_2_O. Thus, urinary bladder compliance improved to 50 mL/cmH_2_O, representing improved LUT function compared to baseline (Fig. 2a). The urinary bladder capacity also increased slightly from 225 mL to 260 mL compared to baseline (Table 2). NBSS showed improved storage and voiding function after 30 sessions of tSCS. There were no changes in the urinary quality of life and incontinence sub-scores (Table 2). The net change in NBSS score was -1, which is not clinically meaningful (the MDC for NBSS total score is -9) (Welk et al. 2014).

**Table 2:** Changes in lower urinary tract function with transcutaneous spinal cord stimulation

Phase	Baseline	Post-30 tSCS sessions
Condition	Without tSCS	Without tSCS
First sensation of urinary bladder filling (mL)	85	205
Strong desire to void / empty urinary bladder (mL)	170	Not Detected
Presence of NDO	Yes (phasic and terminal)	Yes (terminal)
Volume at the first NDO (mL)	100	200
Δ detrusor pressure until first NDO (mL)	8	4
Maximum cystometric capacity (mL)	175	205
Urinary bladder capacity (mL)*	225	260
Maximum change in detrusor pressure (cmH_2_O)	44	41
Urinary bladder compliance (mL/cmH_2_O)	10	50
Presence of urinary leakage	Yes	Yes
Resting BP (HR) before filling cystometry	99/56 mmHg (58 bpm)	101/58 mmHg (52 bpm)
Maximum BP (HR) during filling cystometry	155/91 mmHg (46 bpm)	142/80 mmHg (53 bpm)
NBSS Incontinence	10	10
NBSS Storage and Voiding	15	12
NBSS Consequences	11	13
NBSS Urinary QoL	2	2

Abbreviation: BP = Blood Pressure; HR = Heart Rate; NBSS = Neurogenic Bladder Symptom Score; NDO = Neurogenic detrusor overactivity; QoL = Quality of Life.*Due to the participant could not void voluntarily, urine volume was obtained using a catheter after each filling cystometry.

### Bowel function

At baseline, real-time tSCS induced a consistent increase in anal resting pressure, reaching a maximum with tSCS intensity at 104 mA (Fig. 2b). Unlike the immediate pressure increases observed during 30-second bursts of real-time tSCS, prolonged tSCS at any intensity (> 1 min) resulted in returning anal resting pressure to the baseline level and stabilization of anal resting pressure. The rectoanal inhibitory reflex was present at the baseline assessment (Fig. 2c).

After 30 tSCS sessions, anal resting pressure decreased to 73 mmHg (-38 mmHg from baseline) (Table 3). While there was minimal change in squeeze pressure, thresholds for first sensation and urge to defecate increased. The rectoanal inhibitory reflex remained present after the intervention, with a minimal change in its duration from 16.5 to 17.3 s. Anal sphincter pressure during balloon inflation stimulation of the reflex was reduced from 63 mmHg to 42 mmHg (Fig. 2c). The neurogenic bowel dysfunction score and bowel management method remained unchanged while the time for bowel management was slightly reduced (Table 3).

**Table 3:** Changes in anorectal, bowel and sexual function with transcutaneous spinal cord stimulation

Phase		Baseline	Post-30 tSCS sessions
Condition		Without tSCS	Without tSCS
Resting Pressure (mmHg)		111	73
Squeezing Pressure (△ from Resting Pressure, mmHg)		+ 16	+ 13
First Sensation (mL)		60	90
Urge Sensation (mL)		110	120
Recto-Anal Inhibitory Reflex (RAIR)		Yes	Yes
RAIR Duration (seconds)		16.5	17.3
RAIR Reduced Pressure (mmHg)		63	42
RAIR Amplitude Reduction (△ from Resting Pressure)		43% (48 mmHg)	43% (31 mmHg)
Resting BP (HR)		100/59 mmHg (61 bpm)	97/49 mmHg (55 bpm)
Maximum BP (HR) during anorectal manometry study		126/56 mmHg (54 bpm) during the probe insertion	112/58 mmHg (52 bpm) during the balloon inflation
Time Needed for Bowel Management (minutes)		37	31
Neurogenic Bowel Dysfunction Score (severity of score)		8 (minor)	8 (minor)
IIEF-15	Erectile Function	14	19
	Orgasmic Function	9	5
	Sexual Desire	10	10
	Intercourse Satisfaction	0	14
	Overall Satisfaction	7	9
	Total	40	57

Abbreviation: RAIR = Recto-Anal Inhibitory Reflex; QoL = Quality of Life, SBP = Systolic Blood Pressure, IIEF-15 = International Index of Erectile Function-15 Questionnaire

### Sexual function

At baseline, the participant experienced mild to moderate erectile dysfunction, with a score of 14 out of 30 on the IIEF-15 questionnaire (Table 3). The baseline sexual function was achieved with Cialis™ 5 mg use as needed. Following 30 sessions of tSCS, the participant reported improvements in erectile function, with an increase of + 5 points in the erectile function domain. There was no change in medication or medication frequency. While the orgasmic function score decreased, improvements were observed in the intercourse satisfaction domain, which increased from 0 to 14, and the overall satisfaction domain, which increased from 7 to 9. The total IIEF score, including all domains, increased by + 17 points, exceeding the minimal clinically important difference (MCID) of + 4 (Rosen et al. 2011).

The semi-structured interview demonstrated more detailed changes. Following 30 tSCS sessions, the participant experienced more frequent reflexogenic erections, “*I could just be at the store or something*,* and I looked down and it’s there… kind of like I’m in high school again*.” These reflexogenic erections occurred shortly after tSCS sessions, “*almost every time on my way home*”, and lasted around 20 min. Erection maintenance, however, remained challenging and was managed with the concurrent use of Cialis™ 5 mg prn. The consistent occurrence of erections after tSCS sessions suggests an immediate effect of tSCS on male erectile function.

The participant did not report any changes in his ability to ejaculate, He did, however, note heightened penile sensation, describing it as localized to, “*on the right part of my scrotum*” and “*almost reaching the base of my penis*”; however, this sensitivity did not translate to a noticeable difference to his orgasm function. The participant reported that he regularly experienced AD following ejaculation and/or orgasm, including multiple prolonged episodes of “*horrible*,* horrible*,* throbbing headache*” after ejaculation since 2019. At the time of the interview after long-term tSCS, the participant also noted a positive, experience-enhancing effect of AD: “*It makes me feel very*,* very good… the ejaculation*,* everything now altogether with the AD and the sensation afterward and the spasm. I would compare that as more intense than the orgasms before my injury.*” The improved orgasm was not able to be detected by the IIEF-15 questionnaire. The participant did not describe any significant changes to AD after sexual activities following tSCS, despite reporting fewer bladder-related AD events in the interview. Finally, the participant’s sexual drive remained consistently high throughout and after the tSCS protocol.

### Cardiovascular function

After 30 sessions of tSCS, elevation in SBP during filling cystometry was slightly mitigated compared to the baseline (change in SBP: baseline + 56 mmHg vs. post-intervention + 46 mmHg) (Table 2). During anorectal manometry, while AD was observed at baseline, AD was prevented after long-term tSCS (change in SBP: baseline + 26 mmHg vs. post-intervention + 15 mmHg) (Table 3). During both procedures, bradycardia was observed at baseline, but heart rate showed minimal changes after the intervention (change in heart rate: baseline − 12 bpm vs. post-intervention + 1 bpm during filling cystometry; baseline– 7 bpm vs. post-intervention − 3 bpm during anorectal manometry).

### Neurological level and lower extremity motor function

At baseline, the ISNCSCI without tSCS showed absent sensory and motor responses below T8, except for the left S4-5 dermatome which showed altered pinprick sensation. There was no evidence of voluntary anal contraction or deep anal pressure sensation.

Following 30 sessions of tSCS, the ISNCSCI revealed no change in lower extremity motor function without real-time tSCS. There was, however, partial restoration of sensation in the trunk below T8, the lower extremities (total sensory score + 56 points from baseline), and deep anal pressure sensation without tSCS (Table 4). Despite these sensory improvements, the neurological level of injury and the AIS classification remained unchanged at C4 and AIS B, after the intervention without real-time tSCS. Real-time tSCS further improved sensory score (+ 14 points compared to the absence of tSCS) and lower extremity score (+ 10 points compared to the absence of tSCS), resulting in the AIS conversion to C.

**Table 4:** Neurological examination with international standards for neurological classification of SCI

Phase	Baseline	Post-30 tSCS sessions	
	Without tSCS	Without tSCS	With tSCS at 130 mA
NLI	C4	C4	C4
AIS	B	B	C
Upper Extremity Motor Score	20	19	19
Lower Extremity Motor Score	0	0	10
Light Touch Sensory Score	28	61	66
Pin Prick Sensory Score	21	44	53
Voluntary Anal Contraction	No	No	No
Deep Anal Pressure	No	Yes	Yes

Abbreviation: AIS = American Spinal Injury Association Impairment Scale, C = Cervical, NLI = Neurological level of injury, SCI = spinal cord injury, tSCS = transcutaneous spinal cord stimulation

**Fig. 3:**
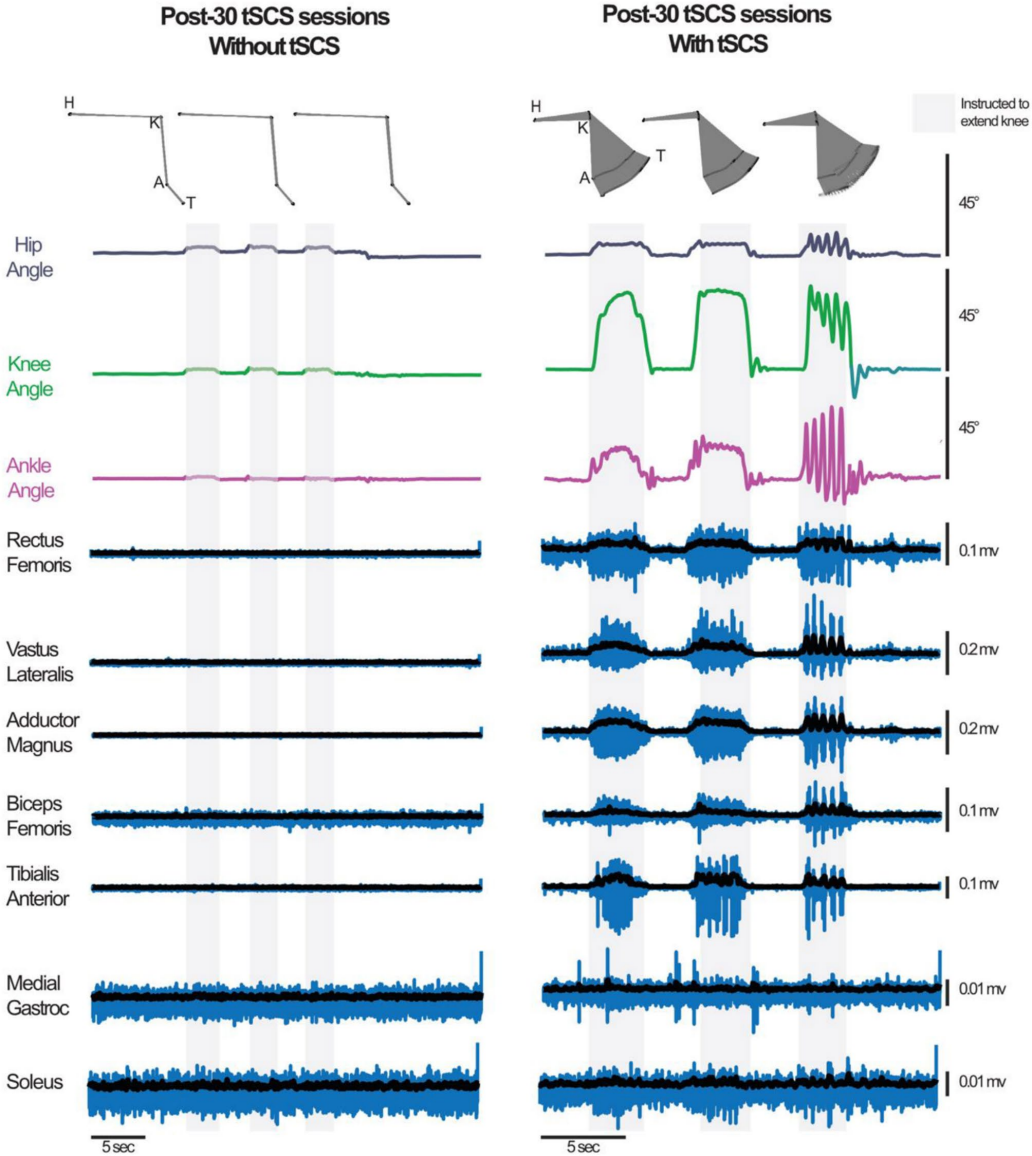
Lower extremity kinematics and muscle activity with and without real-time tSCS post-30 tSCS sessions. Following 30 tSCS sessions, we evaluated the real-time effects of tSCS on lower limb function using kinematic analyses (stick figure representations and joint angles at the hip, knee, and ankle) and electromyography (EMG) recordings of muscle activity. The left panel illustrates kinematics and muscle activity without stimulation, while the right panel demonstrates these outcomes with real-time tSCS applied. Shaded regions represent periods during which the participant was prompted to extend the knee and dorsiflex the ankle, while non-shaded regions correspond to periods when the participant was instructed to relax. These assessments highlight the immediate impact of real-time tSCS on voluntary anti = gravity lower limb movements and muscle activation patterns

In the fourth session of tSCS therapy, the participant noticed the ability to voluntarily contract the right quadriceps muscles for the first time since the onset of SCI. This motor response was only present with real-time tSCS. These movements were eventually observed bilaterally and became more refined and controlled with continued tSCS sessions. Given these observations, we used kinematic and EMG recordings at the post-30 tSCS assessment to quantify return of volitional leg control against gravity in the presence of real-time tSCS (Fig. 3; Video S1). Figure 3 shows attempted contract-relax cycles of combined right knee extension and right ankle dorsiflexion without (**left panel**) and with real-time tSCS (**right panel**) in a seated position. Enabling voluntary control of lower extremity muscles was evidenced by changes in lower extremity joint angles that coincided with bursts of EMG activity (Fig. 3). The average changes in hip, knee, and ankle range of motion during attempted contract-relax cycles were 5.01 ± 2.81 degrees, 22.80 ± 1.71 degrees, and 10.80 ± 6.41 degrees, respectively (Fig. 3). Further, EMG activity during these attempted contract-relax cycles exceeded the resting muscle activity of rectus femoris, vastus lateralis, adductor magnus, biceps femoris, and tibialis anterior by a maximum of 4.09 ± 0.20 SDs, 8.83 ± 1.18 SDs, 15.88 ± 2.30 SDs, 3.77 ± 1.52 SDs, and 12.10 ± 3.90 SDs, respectively. The medial gastrocnemius and soleus muscles did not exceed the resting activity during the trials. In the absence of tSCS there were no changes in joint angles or EMG activity.

## Discussion

Thirty sessions of non-invasive spinal cord stimulation targeting the lumbosacral spinal cord were tolerated well in an individual with chronic motor-complete cervical SCI. Following long-term tSCS, the participant presented with improved LUT, bowel, sexual, and cardiovascular function, alongside the return of voluntary lower extremity movement against gravity.

Long-term tSCS improved multiple pelvic organ function in the participant. Filling cystometry revealed a delayed onset of NDO, resulting in improved urinary bladder compliance, after long-term tSCS, aligned with the previous studies (Kreydin et al. 2020; Gad et al. 2018). Long-term tSCS consistently mitigated the severity of AD during both filling cystometry and anorectal manometry procedures. These findings were reported in the studies with invasive epidural spinal cord stimulation previously (Samejima et al. 2023; Herrity et al. 2022). There is no direct evidence for the mechanisms yet (Samejima et al. 2024). Regarding bowel function, the anal resting pressure was high at baseline compared to the reported healthy control values but after 30 sessions of tSCS, the anal resting pressure was lowered toward the normal values. Furthermore, the relaxation of anal sphincter muscles during rectoanal inhibitory reflex improved after long-term tSCS, which might facilitate the evacuation of stool during bowel management. This is the first report of anorectal manometry testing the effect of long-term tSCS on the anorectal function after motor-complete SCI. These observations suggest that persistent lumbosacral tSCS potentially improved anorectal function. In addition to the spinal cord circuits, the enteric nervous system along with the intestine may also contribute to neuroplastic changes related to bowel function following tSCS (Hamilton et al. 2024; Lefèvre et al. 2023). Further studies are warranted to confirm the efficacy of tSCS for LUT, bowel and cardiovascular function after SCI.

Sexual dysfunction following SCI results from impaired crosstalk between the brain and the spinal reflex arcs of T10-L2 and S2-S4 for sexual function. The disrupted brain-spinal cord communication impairs psychogenic and reflexogenic arousal accompanied by uncontrolled BP elevations after SCI (Sipski et al. 1997, 2006). Following 30 tSCS sessions, the participant reported improved erectile function and genital sensation, which likely contributed to his greater intercourse satisfaction with sexual activity with his partner. These results are similar to studies using implanted lumbosacral epidural spinal cord stimulation (Shackleton et al. 2023; Kandhari et al. 2022; Darrow et al. 2019). The previous studies have demonstrated improvements in sexual function, including decreased sexual distress, increased desire, and improved ejaculation, without and with activity-based training in female and male participants with SCI. Furthermore, we did not have BP measurements during private sexual activity. Based on the semi-structured interview, AD became a positive factor in the orgasmic experience after the intervention phase. Improved cardiovascular control with long-term tSCS might lead to a reduction of AD symptoms. These mild to moderate AD symptoms could improve orgasmic quality, which was seen in the previous study (Courtois et al. 2008). To advance interventions for this understudied aspect of SCI, future research is needed, including preclinical studies and the quantification of key parameters such as erectile function, ejaculation, orgasmic sensation and sperm quality.

The modulation of the lumbosacral spinal cord through tSCS holds potential for inducing positive neuroplastic changes in the spinal cord (Fig. 1) (Wecht et al. 2021; Groat et al. 2015; Browning and Travagli 2014; Sayenko et al. 1985; Krassioukov and Weaver 1996). After SCI, the disrupted communication between the brain and the spinal cord below the injury level results in adverse neural reorganization in the neural pathways and spinal cord circuits, including aberrant plasticity, inflammation, and synaptic changes with loss of supraspinal control (Phillips and Krassioukov 2015; Hou and Rabchevsky 2014; Darian-Smith 2009). The activation of spinal interneurons with tSCS may impact spinal cord excitability (Squair et al. 2021; Samejima et al. 2024). Furthermore, modulation of signal transmission through the gate control by tSCS possibly changes the response to the peripheral stimulation such as bladder distension (Samejima et al. 2023; Melzack and Wall 1965; Kuo et al. 2023). The concurrent functional changes in multiple systems following long-term tSCS indicate neuroplastic changes, including downregulation of calcitonin gene–related peptide-dominant fibers, across the multiple spinal cord segments (Samejima et al. 2024; Elkelini et al. 2012).

In addition to improving pelvic organ functions, the participant showed the increased ISNCSCI sensory score after 30 tSCS sessions. While we did not exam the effect of real-time tSCS on voluntary control in lower extremities, we observed that real-time tSCS enabled bilateral volitional leg control against gravity in a seated position after long-term tSCS. These findings following long-term lumbosacral tSCS align with previous evidence supporting the restoration of motor functions with epidural spinal cord stimulation in individuals with motor-complete SCI (Rowald et al. 2022; Angeli et al. 2018; Darrow et al. 2019). It is important to note that the voluntary lower extremity movements with real-time tSCS were not of the magnitude where functional improvements in daily living would be expected. Nonetheless, even without any advanced closed-loop system or spatiotemporal control, the non-invasive tonic activation of the shared spinal cord segments could be a clinically viable neuromodulatory strategy for multi-modal functional recovery.

The tSCS parameters that we used in this study were based on previous studies primarily targeting sensorimotor function. Similar to tSCS for somatic response, we hypothesize that tSCS current via afferent fibers induces somato-autonomic reflex to modulate autonomic function (Samejima et al. 2024). Somato-autonomic reflex was originally proposed using cutaneous stimulation (Sato et al. 1997). It is still controversial that stimulation sites need to be on the spinal cord, skin or both to induce the modulation of spinal cord excitability via afferent fibers (Lieu et al. 2024). Based on electrophysiological evidence, tSCS with a 10 kHz carrier frequency does not introduce changes in spinal cord excitability compared to conventional tSCS (Gawne et al. 2025; Dalrymple et al. 2023). tSCS with a 10 kHz carrier frequency requires significantly more current to activate the spinal neural structures to elicit comparable reflex responses in neurological conditions (Yang et al. 2025). There is no computational modeling evidence demonstrating the effect of the carrier frequency of tSCS on activating neural structures after SCI. Refinement of stimulation parameters and locations for specific systems needs further inquiry to optimize the potential of tSCS.

Overall, our results highlight the integrated effects of tSCS on multiple functions impacting quality of life in people living with SCI. The multi-modal benefits of bioelectric neuromodulation have been explored using various modalities and neuromodulatory approaches (Moreno Romero et al. 2024). Intensive activity-based training, such as 80 daily sessions of locomotor training with manual assistance, has shown improvements in multiple pelvic organ function after SCI (Hubscher et al. 2018). Sacral nerve root stimulation and laparoscopically implanted neurostimulation, which target peripheral nerves originating from the lumbosacral spinal cord, have demonstrated simultaneous improvements in lower extremity and pelvic organ functions in individuals with SCI (Guiho et al. 2022; Lemos et al. 2023). Similarly, invasive epidural spinal cord stimulation with and without activity-based therapy has shown positive impacts concurrently on lower extremity, cardiovascular and/or pelvic organ functions after SCI (Kandhari et al. 2022; Darrow et al. 2019; Harkema et al. 2011; Barolat et al. 1988; Katz et al. 1991; Sayenko et al. 2019; Beck et al. 2021; Gorgey et al. 2023; Walter et al. 2018). Thus, lumbosacral tSCS holds significant potential for multi-system recovery without surgery (Hubscher et al. 2018, 2021).

### Limitations

This study has several limitations. As a case study from an ongoing clinical trial, it lacks control participants, which limits the ability to draw broader conclusions. The study only involved a total of 30 h of tSCS, which may limit functional gains compared to longer tSCS applications; other epidural spinal cord stimulation studies show improvement with 24-hour application (Darrow et al. 2019) or longer therapy sessions more than 60 sessions (Harkema et al. 2011; Angeli et al. 2014). Although this care report demonstrated functional improvements across multiple systems following long-term tSCS, it did not include evaluations of detailed neurophysiological assessments or neuroanatomical structures. Further research involving a larger sample size is needed to provide mechanistic insight into how long-term tSCS modulates spinal cord circuits.

## Conclusions

This case study demonstrated the multi-modal benefits of lumbosacral tSCS for sensorimotor, cardiovascular, and pelvic organ function in one individual with chronic motor-complete cervical SCI. The observed functional changes suggest that tSCS holds potential for inducing positive therapeutic effects by modulating the nervous system. We anticipate that this non-invasive approach, which does not require intensive fine-tuning by engineers, could evolve into a clinically viable therapy for restoring both motor and autonomic functions in individuals with neurological conditions.

## Electronic supplementary material

Below is the link to the electronic supplementary material.

Supplementary Material 1: **Video S1**: Return of volitional leg control against gravity in the presence of real-time tSCS

## Data Availability

No datasets were generated or analysed during the current study.

## Declaration

### Resource availability

The original contributions presented in the study are included in the article/Supplementary material. Further inquiries can be directed to the corresponding authors.

## Abbreviations

AIS: American Spinal Injury Association Impairment Scale
BP: Blood Pressure
EMG: Electromyography
HR: Heart Rate
ISNCSCI: International Standards for Neurological Classification of Spinal Cord Injury
LUT: Lower Urinary Tract
MCID: Minimal Clinically Important Difference
MDC: Minimal Detectable Change
NBDS: Neurogenic Bowel Dysfunction Score
NBSS: Neurogenic Bladder Symptom Score
NDO: Neurogenic detrusor overactivity
NLI: Neurological level of injury
QoL: Quality of Life
RAIR: Recto-Anal Inhibitory Reflex
SBP: Systolic Blood Pressure
SCI: Spinal Cord Injury
tSCS: Transcutaneous Spinal Cord Stimulation
